# Development of a novel radioligand for imaging 18-kD translocator protein (TSPO) in a rat model of Parkinson’s disease

**DOI:** 10.1186/s12880-019-0375-8

**Published:** 2019-09-18

**Authors:** Chun-Yi Wu, Yang-Yi Chen, Jia-Jia Lin, Jui-Ping Li, Jen-Kun Chen, Te-Chun Hsieh, Chia-Hung Kao

**Affiliations:** 1 Department of Biomedical Imaging and Radiological Science, China Medical University, No.91, Hsueh-Shih Road, Taichung, Taiwan 40402; 2 Master Program for Biomedical Engineering, China Medical University, No.91, Hsueh-Shih Road, Taichung, Taiwan 40402; 3 Department of Biomedical Imaging and Radiological Sciences, National Yang-Ming University, No.155, Sec.2, Linong Street, Taipei, Taiwan 11221; 4 Institute of Biomedical Engineering and Nanomedicine, National Health Research Institutes, 35 Keyan Road, Zhunan, Miaoli County, Taiwan 35053; 5 Graduate Institute of Biomedical Sciences and School of Medicine, College of Medicine, China Medical University, No.91, Hsueh-Shih Road, Taichung, Taiwan 40402; 6 Department of Nuclear Medicine and PET Center, and Center of Augmented Intelligence in Healthcare, China Medical University Hospital, No. 2, Yude Road, North District, Taichung City, Taiwan 40447; 7 Department of Bioinformatics and Medical Engineering, Asia University, 500, Lioufeng Rd., Wufeng, Taichung, Taiwan 41354

**Keywords:** Parkinson’s disease (PD), 18-kD translocator protein (TSPO), [^18^F]FTPQ, Positon emission tomography (PET)

## Abstract

**Purpose:**

The inflammation reaction in the brain may stimulate damage repair or possibly lead to secondary brain injury. It is often associated with activated microglia, which would overexpress 18-kDa translocator protein (TSPO). In this study, we successfully developed a new TSPO radioligand, [^18^F]-2-(4-fluoro-2-(p-tolyloxy)phenyl)-1,2-dihydroisoquinolin-3(4H)-one ([^18^F]FTPQ), and evaluate its potential to noninvasively detect brain changes in a rat model of Parkinson’s disease (PD).

**Procedures:**

The precursor (***8***) for [^18^F]FTPQ preparation was synthesized via six steps. Radiofluorination was carried out in the presence of a copper catalyst, and the crude product was purified by high-performance liquid chromatography (HPLC) to give the desired [^18^F]FTPQ. The rat model of PD was established by the injection of 6-OHDA into the right hemisphere of male 8-week-old Sprague-Dawley rats. MicroPET/CT imaging and immunohistochemistry (IHC) were performed to characterize the biological properties of [^18^F]FTPQ.

**Results:**

The overall chemical yield for the precursor (***8***) was around 14% after multi-step synthesis. The radiofluorination efficiency of [^18^F]FTPQ was 60 ± 5%. After HPLC purification, the radiochemical purity was higher than 98%. The overall radiochemical yield was approximately 19%. The microPET/CT images demonstrated apparent striatum accumulation in the brains of PD rats at the first 30 min after intravenous injection of [^18^F]FTPQ. Besides, longitudinal imaging found the uptake of [^18^F]FTPQ in the brain may reflect the severity of PD. The radioactivity accumulated in the ipsilateral hemisphere of PD rats at 1, 2, and 3 weeks after 6-OHDA administration was 1.84 ± 0.26, 3.43 ± 0.45, and 5.58 ± 0.72%ID/mL, respectively. IHC revealed that an accumulation of microglia/macrophages and astrocytes in the 6-OHDA-injected hemisphere.

**Conclusions:**

In this study, we have successfully synthesized [^18^F]FTPQ with acceptable radiochemical yield and demonstrated the feasibility of [^18^F]FTPQ as a TSPO radioligand for the noninvasive monitoring the disease progression of PD.

**Electronic supplementary material:**

The online version of this article (10.1186/s12880-019-0375-8) contains supplementary material, which is available to authorized users.

## Background

Parkinson’s disease (PD) is a neurodegenerative disorder characterized by impaired dopamine or norepinephrine production, and by the formation of alpha-synuclein. As PD progresses, patients would gradually have difficulty in initiating movement and may have mental and memory problems. Although the real cause of PD remains poorly understood, it is regarded that chronic neuroinflammation plays a vital role for this disease, supported by evidence from activated microglia in the substantia nigra of postmortem brain samples [1, 2] and inflammatory cytokines [3].

Microglia, presenting throughout the central nervous system (CNS), act as the first line of immune defense against invading pathogens and potentially initiate subsequent tissue repair [4]. The activity of microglia in a healthy brain is only at a basal level, but it would be upregulated in response to various CNS damages. The 18-kD translocator protein (TSPO), a transmembrane protein, is previously identified as a peripheral benzodiazepine receptor (PBR), and located in the outer membrane of mitochondria of microglia and astrocytes [5]. TSPO is an essential component of the mitochondrial permeability transition pore (mPTP) and can affect mPTP opening or closure, which would lead to apoptotic death or cell protection. A significantly elevated expression of TSPO has been observed upon the transition of microglia from a normal condition to the activated stage [6]. The glial proliferation may be the reason for the upregulation of TSPO, which can possibly increase neurosteroid synthesis to provide protective activity at injury sites [7]. Moreover, several studies reported that TSPO ligands could serve as a neuroprotective agent in the animal model of neuroinflammation [8–10]. The precise mechanism remains to be fully understood.

[^11^C]PK11195 was the first positron emission tomography (PET) radioligand used to noninvasively quantify the expression level of TSPO in animal models and in PD patients. However, the inconclusive findings were noticed when using [^11^C]PK11195 PET to detect PD. Among these studies, some revealed the accumulation of [^11^C]PK11195 was proportional to the activated expression of TSPO in the brains of PD patients [11, 12], but the others did not support this finding [13]. The low target-to-background ratio of [^11^C]PK11195 caused by low specific binding, high plasma protein binding percentage, and low brain-blood barrier (BBB) penetration ability, might be the explanation for this inconsistency. Besides, the short half-life of C-11 also limits its wide clinical application.

Regarding that TSPO is an appropriate target for noninvasive imaging, a number of second-generation TSPO radioligands, including [^11^C]PBR28, [^11^C]DAA1106, [^18^F]DPA714, and [^18^F]FEPPA, have been developed [14]. Varnas et al. reported that [^11^C]PBR28 accumulation in the brain of PD patients did not correlate with dopaminergic pathology [15]. The inconclusive findings warrant further study to develop novel selective and high-affinity radioactive TSPO ligand for visualization of activated microglia in PD with PET. Based on the scaffold of Ro5–4864, Elkamhawy et al. found the derivatives of 2-(2-aryloxphenyl)-1,4-dihydroisoquinolin-3(2H)-ones are able to modulate the opening/closure of mPTP and suggested them as potential TSPO ligands [16]. To the best of our knowledge, there are no ligands in this class to be labeled with radioisotopes. As a consequence, we synthesized the radioactive surrogate of 2-(2-aryloxphenyl)-1,4-dihydroisoquinolin-3(2H)-ones ([^18^F]FTPQ) and determine its feasibility as a TSPO radioligand for imaging PD in a rat model.

## Methods

### Preparation of the precursor of [^18^F]FTPQ

#### Synthesis of isochroman-3-one (**2**)

**Fig. 1:**
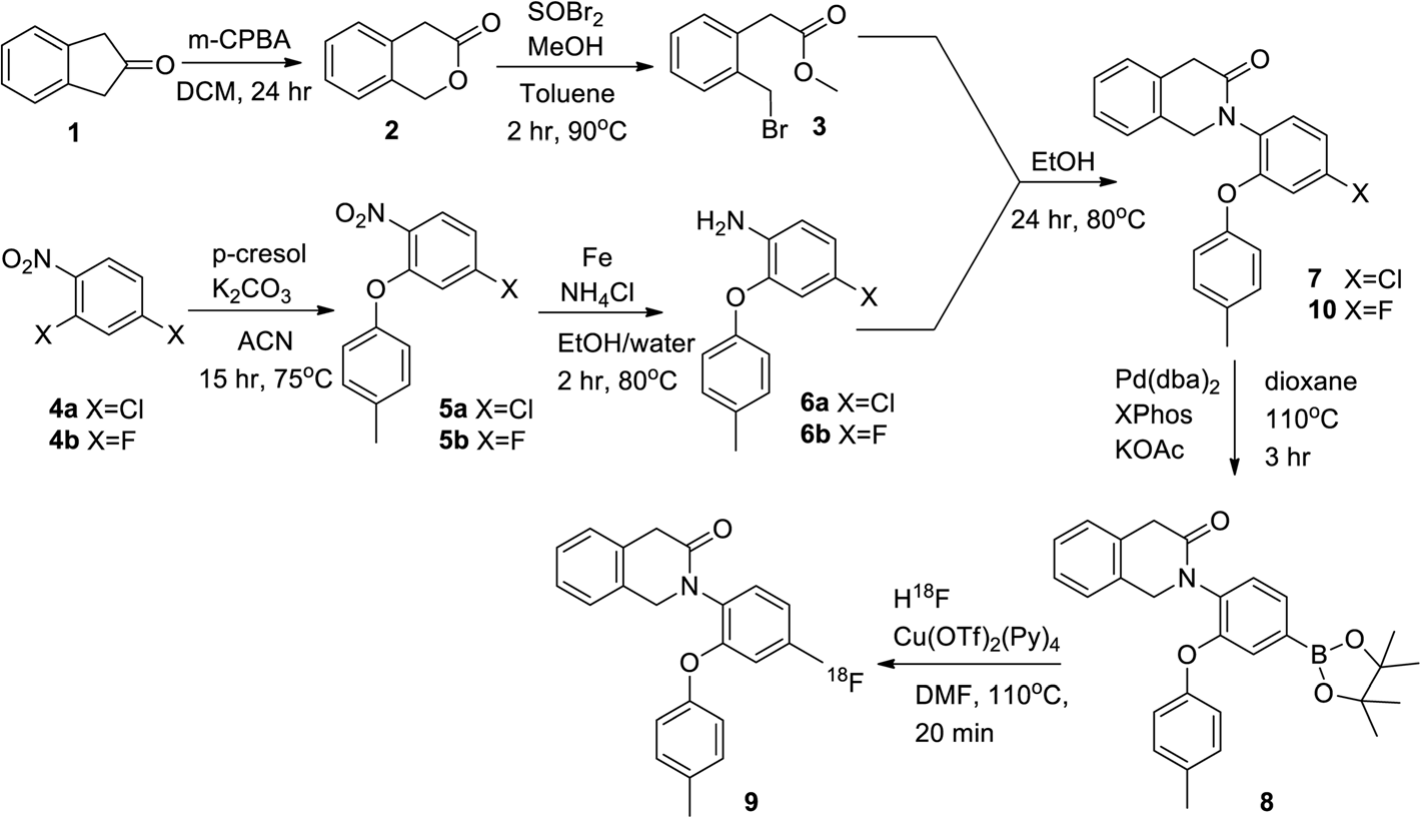
The synthetic scheme of the precursor for radiofluorination (***8***), [^18^F]FTPQ (***8***), and authentic standard (***10***)

The synthetic scheme of the precursor of [^18^F]FTPQ was shown in Fig. 1. In the first step, m-chloroperbenzoic acid (3.92 g, 22.7 mmol) was added into a solution of 2-indanone (***1***) (2 g, 15.13 mmol) in 20 mL of dichloromethane. The reaction mixture was stirred at room temperature (r.t.) for 24 h and then the quenched with 10% aqueous sodium thiosulfate. The resulting mixture was poured into cold brine and extracted with dichloromethane. The organic layer was dried with magnesium sulfate and then evaporated to dryness to give a product (***2***). Yellow solid, yield: 90%, ^1^H NMR (400 Hz, CDCl_3_) δ = 3.70 (2H, s, CH_2_), 5.30 (2H, s, CH_2_), 7.20–7.36 (4H, m, ArH).

#### Synthesis of methyl-2-(2-(bromomethyl)phenyl)acetate (**3**)

To a solution of compound (***2***) (1 g, 6.75 mmol) in methanol (0.9 mL) and anhydrous toluene (30 mL), thionyl bromide (0.71 mL, 9.18 mmol) was dropwise added. The reaction mixture was stirred at 90 °C for 2 h. After the reaction, the resulting mixture was poured into a saturated sodium carbonate solution and stirred for 10 mins. The organic and aqueous layer was extracted with water and dichloromethane, respectively. The combined organic extracts were dried with magnesium sulfate and then evaporated to dryness to give the product (***3***). Brown oil, yield: 50%, ^1^H NMR (400 Hz, CDCl_3_) δ = 3.73 (3H, s, OCH_3_), 3.83 (2H, s, CH_2_), 4.60 (2H, s, CH_2_), 7.29–7.40 (4H, m, ArH).

#### *Synthesis of* 2-chloro-nitro-4(p-tolyloxy)benzene *(****5****)*

2,4-Dicholoronitrobenzene (1 g, 6.29 mmol), 4-methylphenol (680 mg, 6.29 mmol) and potassium carbonate (869 mg, 6.29 mmol) were dissolved in anhydrous acetonitrile (30 mL) and refluxed for 15 h. After the reaction, the solvent was removed under vacco, and the resultant was dissolved in ethyl acetate and then extracted with sodium hydroxide solution (1 N). The organic layer was collected and dried with magnesium sulfate to afford a crude product, which was then purified by column chromatography (hexane/CH_2_Cl_2_ = 4/1) to give the product (***5***). Yellow powder, yield: 64%, ^1^H NMR (400 Hz, CDCl_3_) δ = 2.39 (3H, s, CH_3_), 6.90 (1H, d, *J* = 8.8 Hz ArH), 6.97 (2H, d, *J* = 7.6 Hz, ArH), 7.03 (1H, s, ArH), 7.24 (2H, d, *J* = 7.6 Hz, ArH), 7.96 (1H, d, *J* = 7.6 Hz, ArH).

#### Synthesis of 4-chloro-2-(p-tolyloxy)aniline (**6**)

Compound (***5***) (500 mg, 1.9 mmol) was added into a solution of Fe powder (424 mg, 7.6 mmol) and ammonium chloride (51 mg, 0.95 mmol) in methanol a mixture of EtOH/water (5/2, 7 mL). The reaction mixture was stirred at 80 °C for 1.5 h. After the reaction, the mixture was concentrated in vacco system. The residue was dissolved by ethyl acetate, and the mixture was extracted with brine. The organic extract was collected and dried by dried with magnesium sulfate to afford the product (***6***). Yellow oil, yield: 90%, ^1^H NMR (400 Hz, CDCl_3_) δ = 2.34 (3H, s, CH_3_), 6.7–6.98 (5H, m, ArH), 6.79 (1H, s, ArH), 7.15–7.16 (2H, d, ArH).

#### Synthesis of 2-(4-chloro-2-(p-tolyloxy)phenyl)-1,2-dihydroisoquinolin-3(4H)-one (**7**)

Compound (***3***) (500 mg, 2.057 mmol) and compound (***6***) (721 mg, 3.085 mmol) were dissolved in ethanol (6 mL). The reaction mixture was reacted at 80 °C for 1 d. After the reaction, the mixture was evaporated in vacco system to dryness, and the crude residue was purified by column chromatography (CH_2_Cl_2_/EA = 10/1) to afford the product (***7***). Yellow solid, yield: 76%, ^1^H NMR (400 Hz, CDCl_3_) δ = 2.30 (3H, s, CH_3_), 3.67 (2H, s, CH_2_), 4.74 (2H, s, CH_2_), 6.88–7.11 (3H, m, ArH), 7.12–7.27 (8H, m, ArH). ^13^C NMR (400 Hz, CDCl_3_) δ = 20.7, 38.2, 53.7, 119.0, 119.5, 123.7, 125.1, 126.7, 127.3, 127.7, 130.3, 130.4, 131.2, 131.8, 132.4, 133.9, 134.1, 153.6, 153.9, 169.4.

#### Synthesis of 2-(4-(4,4,5,5-tetramethyl-1,3,2-dioxaborolan-2-yl)-2-(p-tolyloxy)phenyl)-1,2-dihydroisoquinolin-3(4H)-one (**8**)

Compound (***7***) (1 g, 2.75 mmol), bis(pinacolato)diboron (2.09 g, 8.25 mmol), potassium acetate (810 mg, 8.25 mmol), Pd(dba)_2_(0) (31.6 mg, 0.06 mmol), and 2-dicyclohexylphosphino-2′,4′,6′-triisopropylbiphenyl (52.5 mg, 0.11 mmol) were dissolved in dioxane (5 mL). The mixture was reacted at 110 °C for 3 h. After the reaction, the reaction mixture was concentrated in vacco to remove the solvent. The residue was dissolved in dichloromethane and extracted with brine. The organic layer was collected and dried with magnesium sulfate to afford a crude product, which was then purified by column chromatography (CH_2_CH_2_/EA = 5/1). The product (***8***) was washed with hexane and then recrystallized in a hexane/dichloromethane system. White solid, yield: 73%, ^1^H NMR (400 Hz, CDCl_3_) δ = 1.28 (12H, s, CH_3_), 2.24 (3H, s, CH_3_), 3.58 (2H, s, CH_2_), 4.68 (2H, s, CH_2_), 6.80 (2H, d, *J* = 7.6 Hz, ArH), 7.0 (2H, d, *J* = 8.0 Hz ArH).7.07–7.23 (4H, m, ArH), 7.30 (1H, d, *J* = 7.6 Hz, ArH), 7.47 (1H, s, ArH), 7.61 (1H, d, *J* = 7.6 Hz ArH) ^13^C NMR (400 Hz, CDCl_3_) δ = 20.6, 24.8, 38.3, 53.6, 84.0, 117.6, 125.1, 126.6, 126.9, 127.2, 127.6, 129.0, 130.0, 130.8, 132.1, 132.4, 132.5, 136.2, 151.8, 155.0, 169.2. LRMS (ES+): m/z calculated for C_22_H_19_O_2_NF: 348.1389.

#### Synthesis of the authantic standard (**10**)

Compound (***10***) was prepared according to the method described above, except for 2,4-dicholoronitrobenzene was replaced by 2,4-difluoronitrobenzene. Yellow solid, yield: 73%, ^1^H NMR (400 Hz, CDCl_3_) δ = 2.31 (3H, s, CH_3_), 3.69 (2H, s, CH_2_), 4.76 (2H, s, CH_2_), 6.65 (1H, d, *J* = 10 Hz, ArH), 6.82 (1H, t, *J* = 10 Hz, ArH), 6.91 (2H, d, *J* = 7.6 Hz, ArH), 6.82 (2H, d, *J* = 8 Hz, ArH), 7.14 (1H, d, *J* = 7.2 Hz, ArH), 7.19 (1H, d, *J* = 7.2 Hz, ArH), 7.24–7.28 (3H, m, ArH). ^13^C NMR (400 Hz, CDCl_3_) δ = 20.7, 38.2, 53.9, 106.6, 106.8, 110.2, 110.4, 119.1, 125.1, 126.7, 127.3, 127.7, 130.4, 130.5, 131.9, 132.4, 133.9, 153,6, 154.5, 154.6, 160.1, 163.5, 169.5. HRMS (ES+): m/z calculated for C_22_H_19_O_2_NF: 348.1389.

### Preparation of [^18^F]FTPQ

Aqueous no-carrier-added [^18^F]HF was transferred through a QMA Sep-Pak cartridge conditioned with ethanol (10 mL), KOTf solution (90 mg/mL, 10 mL), and ddH_2_O (10 mL). The fluoride trapped in QMA cartridge was eluted with 0.55 mL of eluent (100 mg of KTOf and 1 mg of K_2_CO_3_ in 11 mL of ddH_2_O) to a V-vial. The solvent was evaporated to dryness at 110 °C, and then anhydrous acetonitrile (1 mL) was added into the vial as an azeotrope. To the dry residue, 3 mg of precursor (***8***) and 0.5 mL of catalyst solution (Cu(II)(OTf)_2_-Pyridine DMF, 36.5 mg of Cu(OTf)_2_ dissolved in 0.2 mL of pyridine and 2.5 mL of DMF) were added. The reaction mixture was reacted at 110 °C for 20 min. After cooling, the reaction mixture was passed through a Plus Silica Sep-Pak cartridge (Waters, USA), which was preconditioned with 10 mL hexane. The crude product trapped in Sep-Pak was eluted with 2 mL of dichloromethane. After evaporation of the solvent under a stream of nitrogen at 110 °C, the mixture was re-dissolved in acetonitrile. The collected crude product was purified by high-performance liquid chromatography (HPLC), which was performed on a reversed-phase column (semipreparative (VP 250/10) NUCLEODUR C18 HTEC, 5 μm) using 70% acetonitrile in ddH_2_O as the mobile phase at a flow rate of 4 mL/min. The desired fraction was collected and then evaporated to dryness for removing acetonitrile. The residue was redissolved with normal saline and then filtered by a 0.22-μm filter (Millex-OR, Millipore). The specific activity of [^18^F]FTPQ was determined using an analytical C-18 HPLC column (NUCLEODUR C18 HTEC, 5 μm) eluted with 70% acetonitrile in ddH_2_O at a flow rate of 1 mL/min.

### Partition coefficient of [^18^F]FTPQ

The partition coefficient of [^18^F]FTPQ was assessed by determining its distribution between 1-octane and phosphate buffer saline (PBS) and expressed as log *P*. To a tube containing 1 mL of 1-octane and 1 mL of PBS, [^18^F]FTPQ (7.4 kBq) was added then the mixture was vortexed for 1 min. After vortexing, the tube was centrifuged at 1000 g for 5 min. Aliquots (500 μL) were taken from the organic phase and added into the next tube. These steps were repeated for five times. Finally, a hundred microliters of each layer were collected for the measurement of radioactivity by a gamma counter (1470 WIZARD Gamma Counter, Wallac, Finland).

### Stability of [^18^F]FTPQ

[^18^F]FTPQ was incubated in either normal saline or fetal bovine serum (FBS) at 37 °C for 30, 60, 120, and 240 min to investigate the in vitro stability, which expressed as the percentage of the intact radioactive compound analyzed by radioTLC.

### Establishment of 6-OHDA-lesioned animal model

The protocol was based on the method published by Weng et al. with some minor modifications [17]. Briefly, male 8-week-old Sprague-Dawley rats, purchased from LASCO Taiwan Co., Ltd. (Yilan, Taiwan), were placed in a stereotaxic device and anesthetized under 2% isoflurane in O_2_. When the heart rate of rats reached a steady state, a small hole located on the right side of the skull (1.2 mm right lateral to and 4.4 mm posterior to the bregma) was created by an electric drill for animal use. Twenty micrograms of 6-hydroxydopamine (6-OHDA, in 4 μL of normal saline containing 0.02% ascorbic acid) were injected into the brain using syringe pump (1 μL/min) on day 0. The injection point was 7.8 mm below the dura. After injection, the syringe was vertically kept for 5 min, and slowly removed from the brain at a rate of 1 mm/min. The hole was covered with a bone flap, and the wound was sealed after administration. The rats in the sham group received the same procedures except for the 6-OHDA injection (*n* = 5).

### Apomorphine-induced rotational behavior test

For a selection of diseased rats, the rats were intraperitoneally injected with apomorphine (5 mg/kg) on day 14 after 6-OHDA administration. The rats were then subjected to a rotational behavior test for at least 100 min. Only those with contralateral rotation > 300 turns/h were used in following imaging experiments (*n* = 5).

### [^18^F]FTPQ microPET imaging

Imaging studies were performed on a microPET scaaner (Inveon PET, Siemens). Static microPET/CT imaging was conducted for 30 min immediately after injection of 18.5 MBq of [^18^F]FTPQ on days 7, 14 and 21 for diseased animals and controls (n≧5). During the examination, the rats were anesthetized with 2% isoflurane in O_2_ and placed in the prone position with the long axis parallel to the table of the scanner. The rats were sacrificed by CO_2_ inhalation immediately after imaging studies for histological analysis to confirm the biological features of PD.

### Histological analysis

After microPET/CT imaging, the PD rats were sacrificed and perfused with 100 mL of phosphate buffer solution. The brains were excised for immunohistochemistry staining to assess the expression levels of the dopaminergic pathway, and the number of microglia and astrocytes. The dehydration, paraffin embedding, and section steps were conducted as previously described [18]. The slices were incubated with 3% H_2_O_2_ for 20 min and then blocked with Protein Block (Abcam) at r.t. for 10 min. Heat-induced antigen retrieval was performed with 0.01 M of citrate buffer (pH = 6.0) at 85 °C for 30 min. The rat monoclonal antibodies against tyrosine hydroxylase (TH, ab112, Abcam), CD68 (mca341r, Bio-Rad), and GFAP (Z0334, Dako) were applied to the slides at a dilution of 1:750, 1:100, and 1:1000, respectively. The slides for TH, CD68, and GFAP staining were exposed to the rabbit specific HRP/DAB detection kit (ab64261, Abcam for TH; S8125, Dako for CD68; SK4600, Vector for GFAP) until the brown or purple stains were observed.

### Statistical analysis

All data were expressed as the mean **±** standard deviation (S.D.). The Student’s *t*-tests were applied for the comparison between groups. Values of *P* < 0.05 were considered as statistically significant.

## Results

### The synthesis of the precursor (8) and authentic standard (10)

After multi-step synthesis, the overall chemical yield for the precursor (***8***) and authentic standard (***10***) was 14 and 6%, respectively. The ^1^H, ^13^C NMR, and mass spectrometry for all compounds were shown in the Additional file 1.

### Preparation of [^18^F]FTPQ

**Fig. 2:**
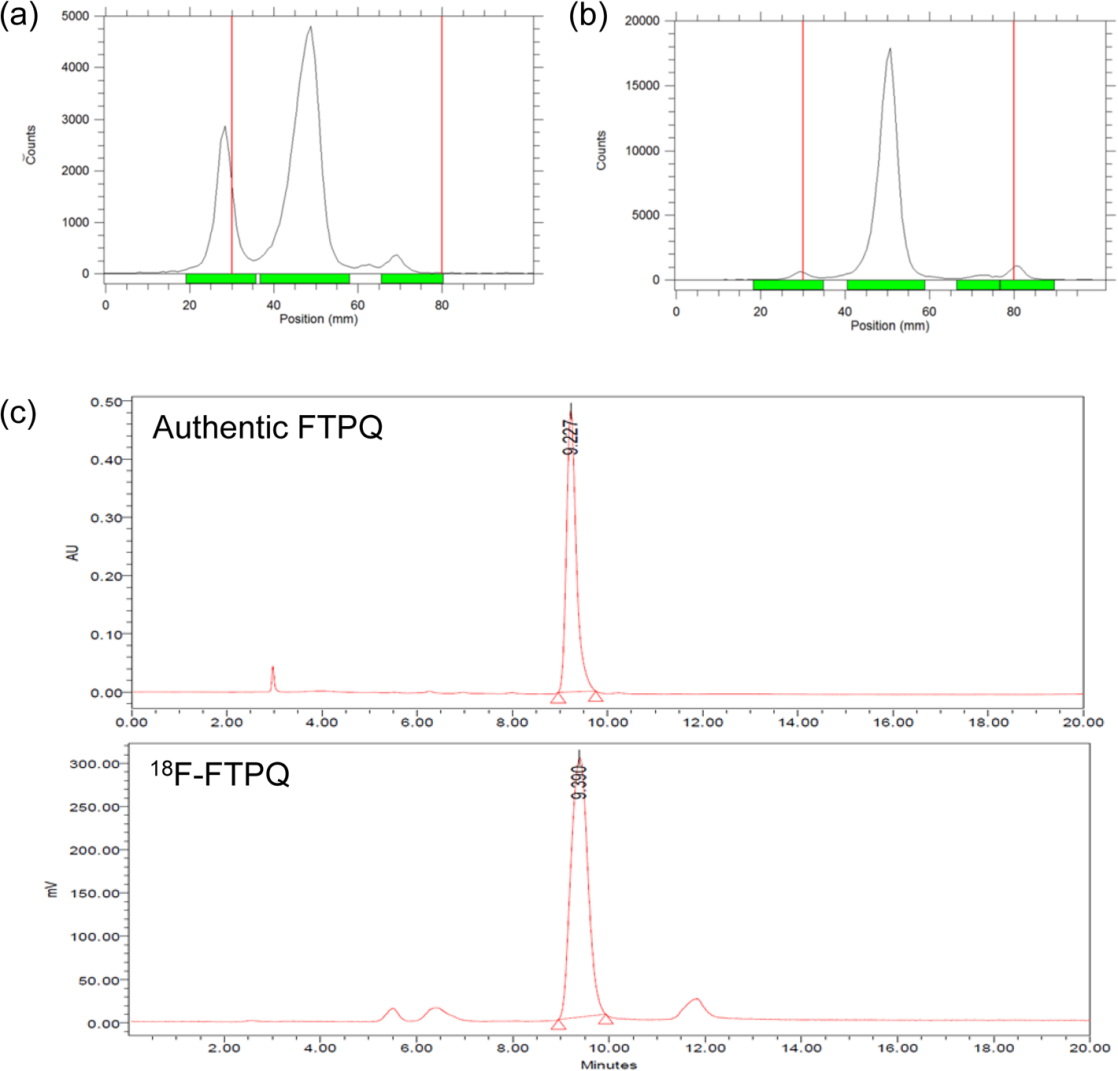
RadioTLC of crude [^18^F]FTPQ (**a**) before and (**b**) after the Sep-pak cartridge purification. **c** HPLC of [^18^F]FTPQ. The retention time of authentic FTPQ and [^18^F]FTPQ was 9.23 and 9.39 min, respectively

The radiofluorination efficiency of [^18^F]FTPQ was around 60 ± 5% (Fig. 2a). After the purification using Sep-Pak cartridge, most of the unlabeled radiofluorine would be removed (Fig. 2b). Increasing reaction time (more than 20 min) did not facilitate the SN2 reactivity to give higher labeling efficiency (data not shown). After HPLC separation, the radiochemical purity of the desired product was greater than 98%. The retention time of authentic standard and [^18^F]FTPQ was 9.227 and 9.390 min, respectively (Fig. 2c). A small difference in the retention time between standard and radioactive ligand originated from the travel time in the connecting loop and suggested radioactive ligand owns the identical structure with that of standard. The total preparation time for [^18^F]FTPQ was approximately 100 min with an overall radiochemical yield of 19%, which was corrected for the physical decay (d.c.). The specific activity of [^18^F]FTPQ, determined by analytic HPLC column, was approximately 1.5 GBq/μmol.

### In vitro stability and partition coefficient of [^18^F]FTPQ

**Fig. 3:**
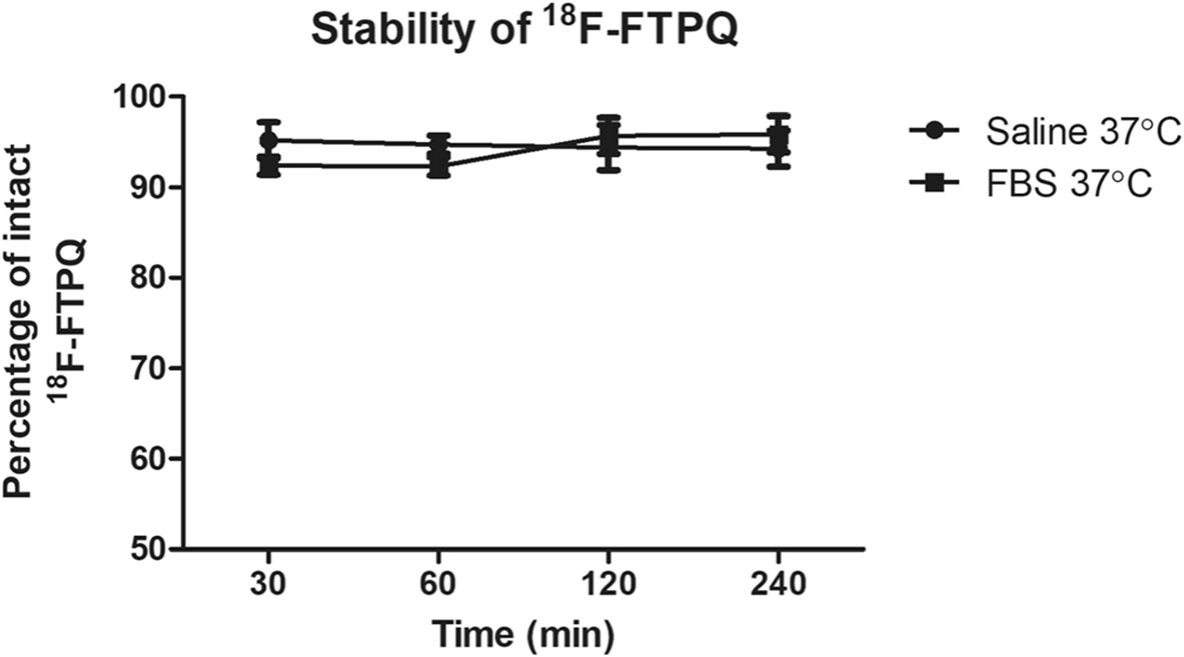
In vitro stability of [^18^F]FTPQ in either normal saline or in FBS at 37 °C

The in vitro stability of [^18^F]FTPQ in either normal saline or FBS at 37 °C was assessed by a radioTLC scanner. The percentage of intact [^18^F]FTPQ was more than 90% in both conditions after 4 h of incubation (Fig. 3). A log *P* value of 1.69 ± 0.16 in n-octanol/water revealed that [^18^F]FTPQ is considerably a hydrophobic compound, which may enhance its penetration through the blood-brain barrier (BBB).

### MicroPET/CT imaging

**Fig. 4:**
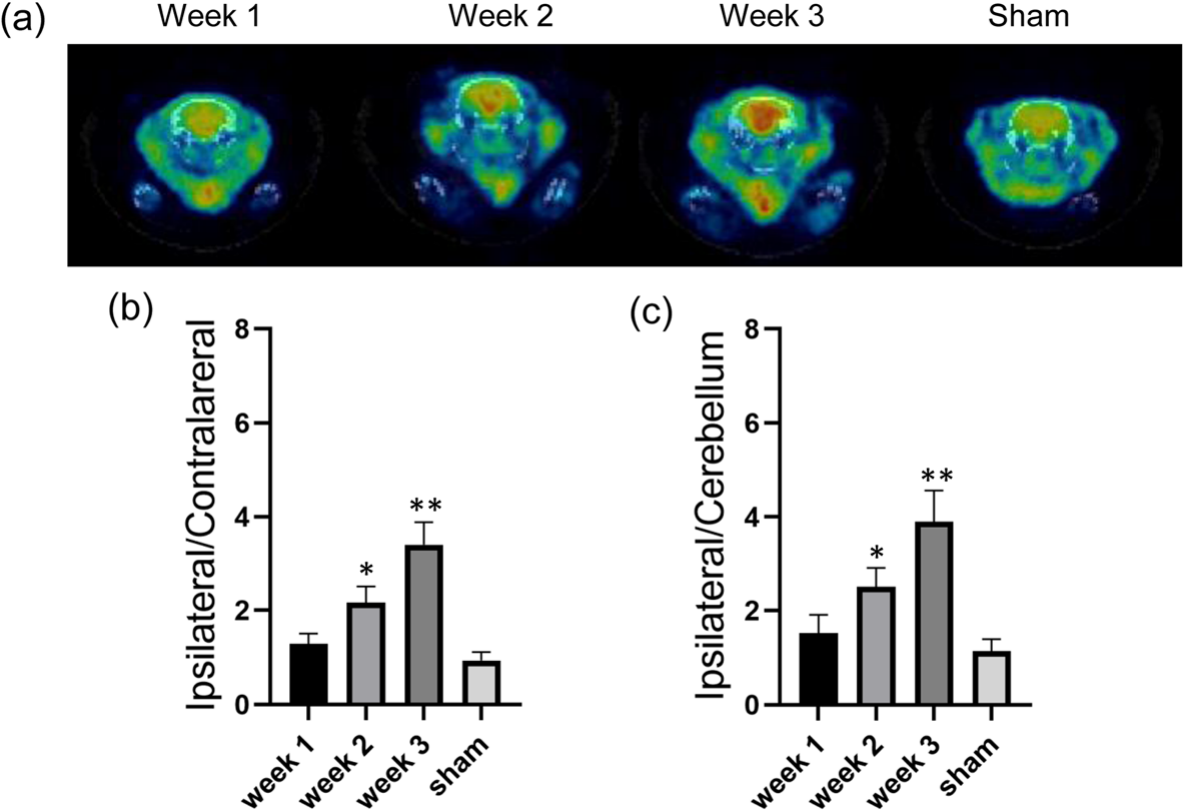
**a** MicroPET/CT imaging of PD rats after intravenous injection of approximately 18.5 MBq of [^18^F]FTPQ for 30 min. The semi-quantitative analysis of (**b**) ipsilateral hemisphere-to-contralateral hemisphere ratio and (**c**) ipsilateral hemisphere-to-cerebellum ratio from the images of the 1-, 2-, and 3-week old PD rats and the sham rats. *significant where *P* < 0.05, **where significant where *P* < 0.001 (PD diseased rats compared with sham groups)

The microPET/CT images demonstrated apparent striatum accumulation in the brains of PD rats (*n* = 5) at the first 30 min after intravenous injection of [^18^F]FTPQ, while the uptake in the sham group (*n* = 5) was not evident (Fig. 4). Longitudinal imaging found the uptake of [^18^F]FTPQ in the brain may reflect the severity of PD. The radioactivity accumulated in the ipsilateral brains of PD rats at 1, 2, and 3 weeks after 6-OHDA administration was 1.84 ± 0.26, 3.43 ± 0.45, and 5.58 ± 0.72%ID/mL, respectively. However, the clearance of [^18^F]FTPQ from the brains of the sham group was more rapid than that of the PD rats, resulting in a relatively low uptake in the brain (1.57 ± 0.29%ID/mL). The ipsilateral semisphere-to-cerebellum ratio derived from the [^18^F]FTPQ mircroPET/CT images of the 1-, 2-, 3-week PD and the control groups was 1.51 ± 0.39, 2.51 ± 0.40, 3.90 ± 0.67, and 1.13 ± 0.26, respectively.

### Histological analysis

**Fig. 5:**
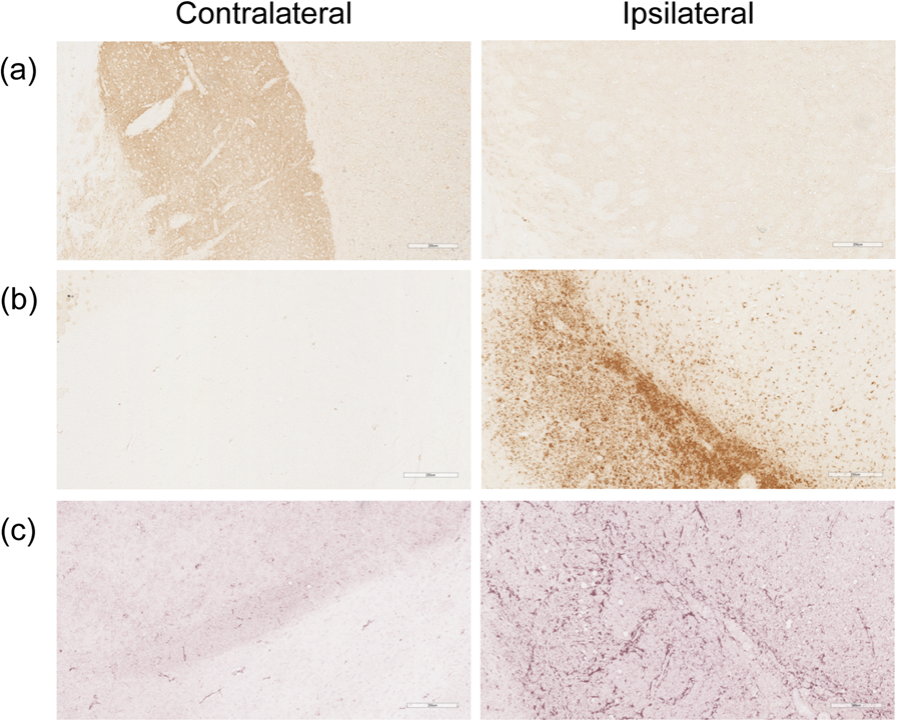
Immunohistochemical staining of the brain sections from PD rats and the controls. The brain was excised from rats to assess the difference in (**a**) tyrosine hydroxylase (TH) expression, (**b**) macrophage activation (CD68), and (**c**) astrocyte activation (GFAP)

Tyrosine hydroxylase (TH) staining was applied to confirm the loss of dopamine neurons in the striatum and substantia nigra of PD rats. In rats received 6-OHDA, the TH stains markedly decreased when compared with that of the sham group (Fig. 5a), suggesting 6-OHDA-treated rats are appropriately considered as those mimicking the pathology in the patients with PD. When compared with the contralateral hemisphere, the 6-OHDA-injected side showed intense CD68 and GFAP staining (Fig. 5b and c), which are considered as markers for pan-macrophage and microglia, and reactive astrocytes index, respectively, suggesting the high accumulation of [^18^F]FTPQ was associated with neuroinflammation in the brains of PD rats.

## Discussion

The 4-chlorodiazepam (Ro5–4864) exhibits nanomolar affinity to TSPO as well as PK11195 in some species [19]. Elakamhawy et al. developed the derivatives of Ro5–4864 via opening its diazepine ring and found some of these compounds are capable of modulating mPTP at nanomolar range [16], suggesting their strong binding affinity to TSPO. Considering no previous study investigated the in vivo distribution of this class of compounds, this study was designed to explore the biological characteristics of a new TSPO ligand, [^18^F]FTPQ. For the synthesis of the precursor (***8***) for radiolabeling, the addition of boron ester on the compound (***7***) is the most critical step. In fact, this reaction would not work if [1,1′-Bis(diphenylphosphino)ferrocene]dichloropalladium (II) (complex with dichloromethane, Pd(dppf)Cl_2_·DCM) was used as a catalyst, suggesting the electron density of the catalyst apparently affects the catalytic activity. For radiolabeling, the reason for the relatively low radiochemical yield actually originated from the poor solubility of [^18^F]FTPQ.

It is suggested that the inflammatory processes of PD vary over time. Therefore, optimal management could benefit from a reliable noninvasive imaging technique that can reflect disease progression or severity. The 6-OHDA-lesioned rat model is regarded as an appropriate animal model for preclinical studies. Maia et al. discovered that the in vitro accumulation of [^3^H]PK11195 in the ipsilateral striatum on day 21 post-lesion (p.l.) was significantly lower than that on day 7 p.l. [20]. The ex vivo binding of ^125^I-CLINDE to the ipsilateral striatum was similar on days 7 and day 14 p.l. but decreased apparently on day 21 p.l. [20]. Vetel et al. also found the specific retention of [^3^H]DPA-714 in the ipsilateral striatum was significantly higher than that in the contralateral striatum on day 14 p.l. [21]. Besides, previous clinical PET imaging using [^11^C]PK11195 revealed a high uptake in the brain of PD patients due to the activated microglia [11, 12]. However, this finding was not conclusive since some groups reported dramatically different results that did not observe significant radioactivity retained in the PD brains [13]. Terada et al. demonstrated similar elevated TSPO binding in the PD patients’ brains when using [^11^C]DPA-713 as a radioligand for the assessment [22], but not in the investigations using [^18^F]FEPPA performed by Koshimori et al. [23] and Ghadery et al. [24].

The imaging results of [^18^F]FTPQ in this study corroborate previous findings with [^11^C]PK11195 [11, 12] and [^11^C]DPA-713 [22] showing enhanced brain accumulation in PD rats (Fig. 4). In addition, the uptake of [^18^F]FTPQ in PD brains increased over the experimental period, suggesting the radioactivity accumulation can be a quantification index for monitoring PD activity (Fig. 4) although the profile was inconsistent with the previous [^11^C]PK11195 autoradiography results, which demonstrated the maximum TSPO expression occurred on day 7 p.l. [20]. The possible explanations for this discrepancy are the difference in binding affinity between [^18^F]FTPQ and [^11^C]PK11195, and the autoradiography findings were obtained from in vitro experiments rather than from in vivo. In fact, the lower log *P* value of [^18^F]FTPQ than that of PK11195 can account for its reduced non-specific binding in the brain of the sham group (Fig. 4). Similar to previous studies [25, 26], immunohistochemical staining for CD68 and GFAP revealed that brains from 6-OHDA-lesioned PD rats have a significantly increased number of microglia/macrophages and astrocytes compared to the contralateral sites (Fig. 5).

As mentioned above, [^18^F]FTPQ shares a similar scaffold with Ro5–4864, which binds to TSPO in a temperature- and species-dependent manner [27]. In addition, a single-nucleotide polymorphism, rs6917, has been confirmed as a critical factor affecting the binding affinity of second-generation TSPO radioligand and causing inter-individual variability [28]. Further experiments are warranted to clarify these issues.

## Conclusions

In this study, we have successfully synthesized a new TSPO radioligand, [^18^F]FTPQ, with acceptable radiochemical yield and demonstrated that the accumulation of [^18^F]FTPQ in brain may be a useful index for the detection of PD and monitoring the disease progression. To our best knowledge, this is the first study to determine the in vivo pharmacokinetics of this new class of TSPO ligand through noninvasive imaging technique.

## Additional file

Additional file 1: All spectral data of compound 1-10. (DOCX 2913 kb)

## Data Availability

Data sharing is not applicable to this article as no datasets were generated or analysed during the current study.

## Abbreviations

[^18^F]FTPQ: [^18^F]-2-(4-fluoro-2-(p-tolyloxy)phenyl)-1,2-dihydroisoquinolin-3(4H)-one
BBB: Brain-blood barrier
CNS: Central nervous system
FBS: Fetal bovine serum
HPLC: High-performance liquid chromatography
IHC: Immunohistochemistry
mPTP: Mitochondrial permeability transition pore
PBR: Peripheral benzodiazepine receptor
PBS: Phosphate buffer saline
PD: Parkinson’s disease
PET: Positron emission tomography
TH: Tyrosine hydroxylase
TSPO: Translocator protein
