# Supplementary material for: Development of a novel radioligand for imaging 18-kD translocator protein (TSPO) in a rat model of Parkinson’s disease
Source: BMC Med Imaging. 2019 Sep 18;19:78. doi: 10.1186/s12880-019-0375-8 (PMC6751751; doi:10.1186/s12880-019-0375-8)

***Molecular Imaging and Biology***

**Supporting Information**

**Development of a novel radioligand for imaging 18-kD translocator protein (TSPO) in a Rat Model of Parkinson’s Disease**

Chun-Yi Wu^1,2^, Yang-Yi Chen^3^, Jia-Jia Lin^1^, Jui-Ping Li^4^, Jen-Kun Chen^4^, *Te-Chun Hsieh^1,5^, *Chia-Hung Kao^5,6,7^

^1^Department of Biomedical Imaging and Radiological Science, China Medical University, Taichung, Taiwan

^2^Master Program for Biomedical Engineering, China Medical University, Taichung, Taiwan

^3^Department of Biomedical Imaging and Radiological Sciences, National Yang-Ming University

^4^Institute of Biomedical Engineering and Nanomedicine, National Health Research Institutes, Miaoli, Taiwan

^5^Graduate Institute of Biomedical Sciences, College of Medicine, China Medical University, Taichung, Taiwan

^6^Department of Nuclear Medicine and PET Center, China Medical University Hospital, Taichung, Taiwan

^7^Department of Bioinformatics and Medical Engineering, Asia University, Taichung, Taiwan

***Correspondence:**

Chia-Hung Kao

No.2 Yude Rd., North Dist., Taichung 40447, Taiwan.

Tel: +886-4-22052121 ext. 7412

Fax: +886-4-22336174

Email: [d10040@mail.cmuh.org.tw](mailto:d10040@mail.cmuh.org.tw)

Te-Chun Hsieh

No.2 Yude Rd., North Dist., Taichung 40447, Taiwan.

Tel: +886-4-22052121 ext. 7412

Fax: +886-4-22336174

Email: [rancholosamigos@mail.cmuh.org.tw](mailto:rancholosamigos@mail.cmuh.org.tw)

The content of supporting information includes all spectral data of compound **1-10**.

*isochroman-3-one (****2****)*


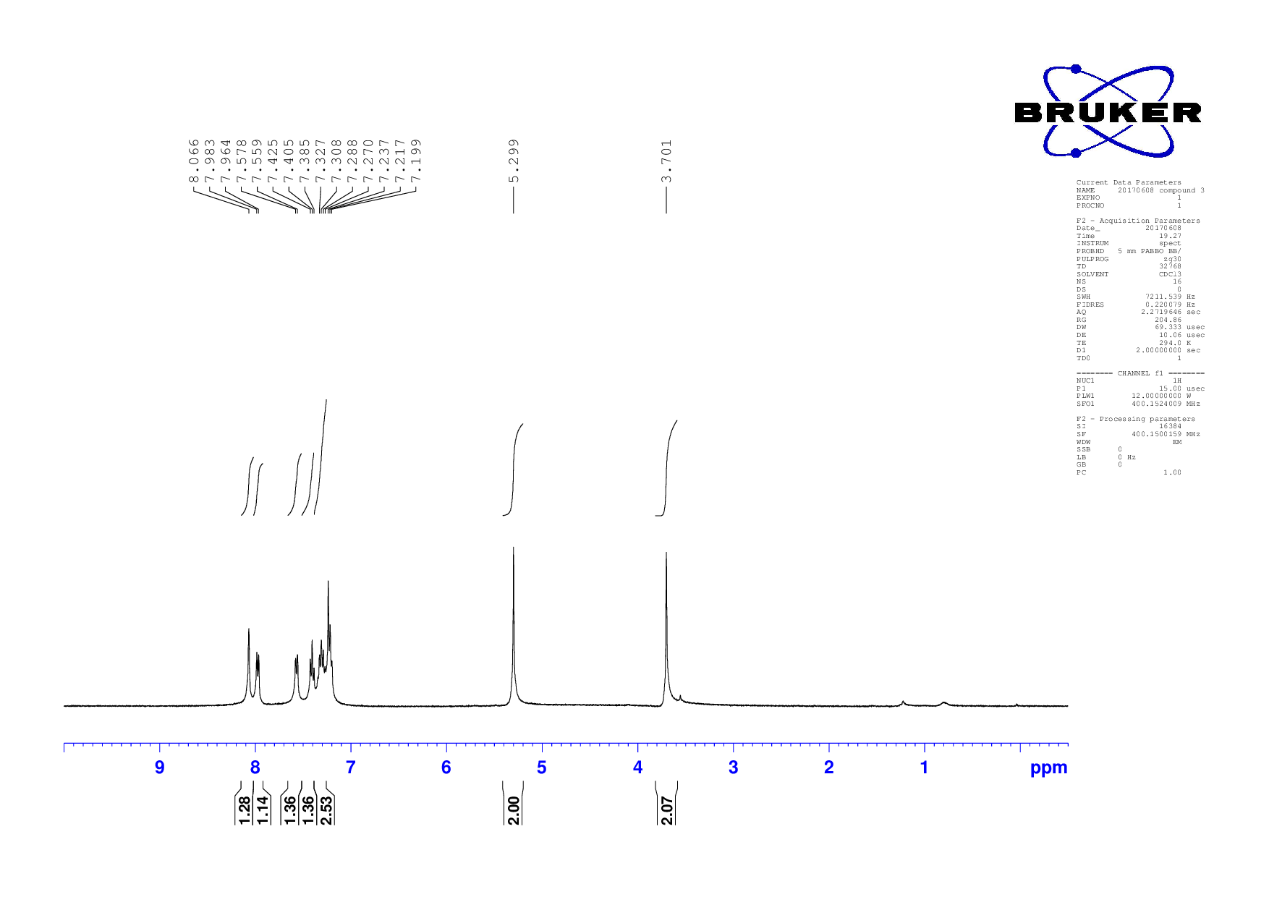


*methyl-2-(2-(bromomethyl)phenyl)acetate (****3****)
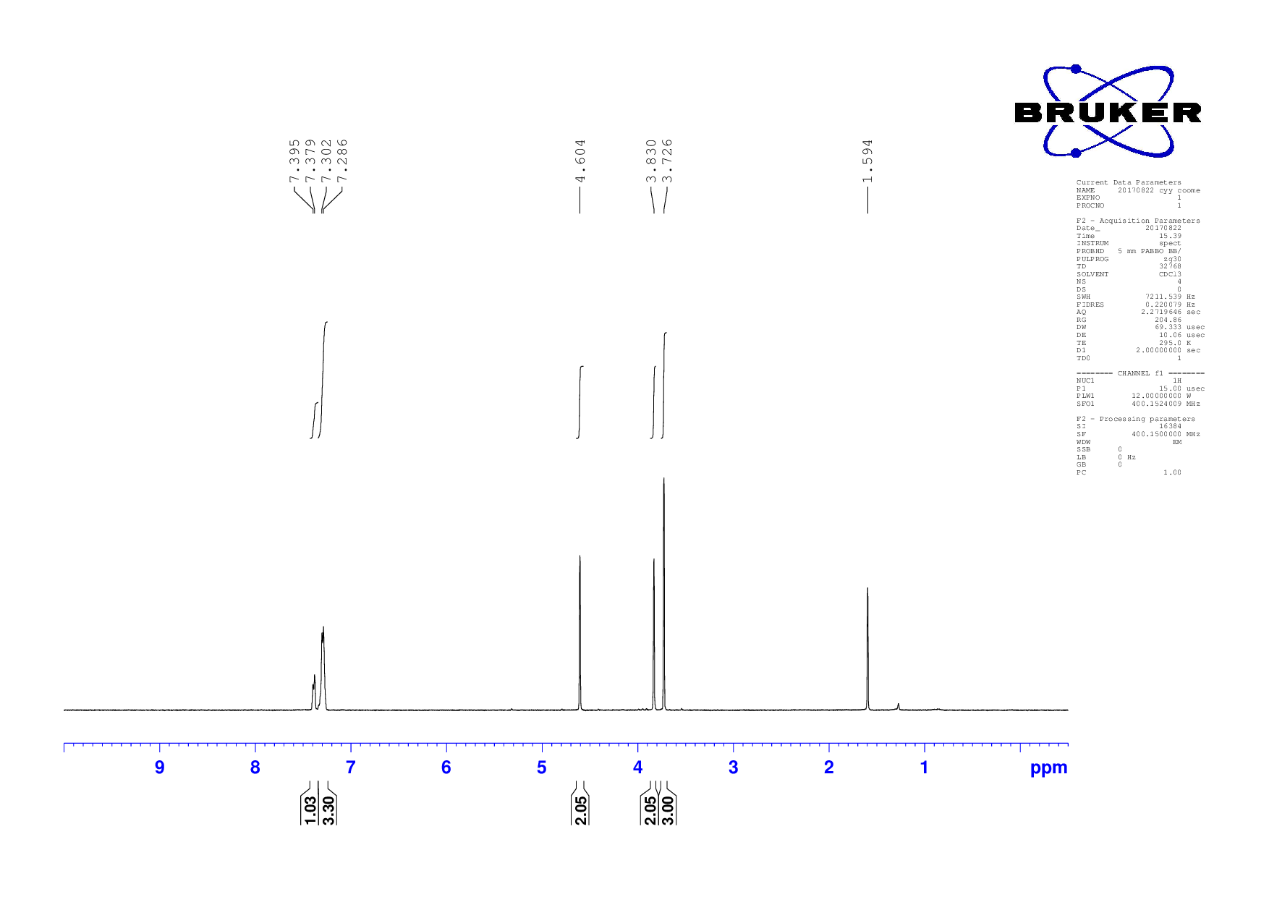
*

2-chloro-nitro-4(p-tolyloxy)benzene *(****5a****)*


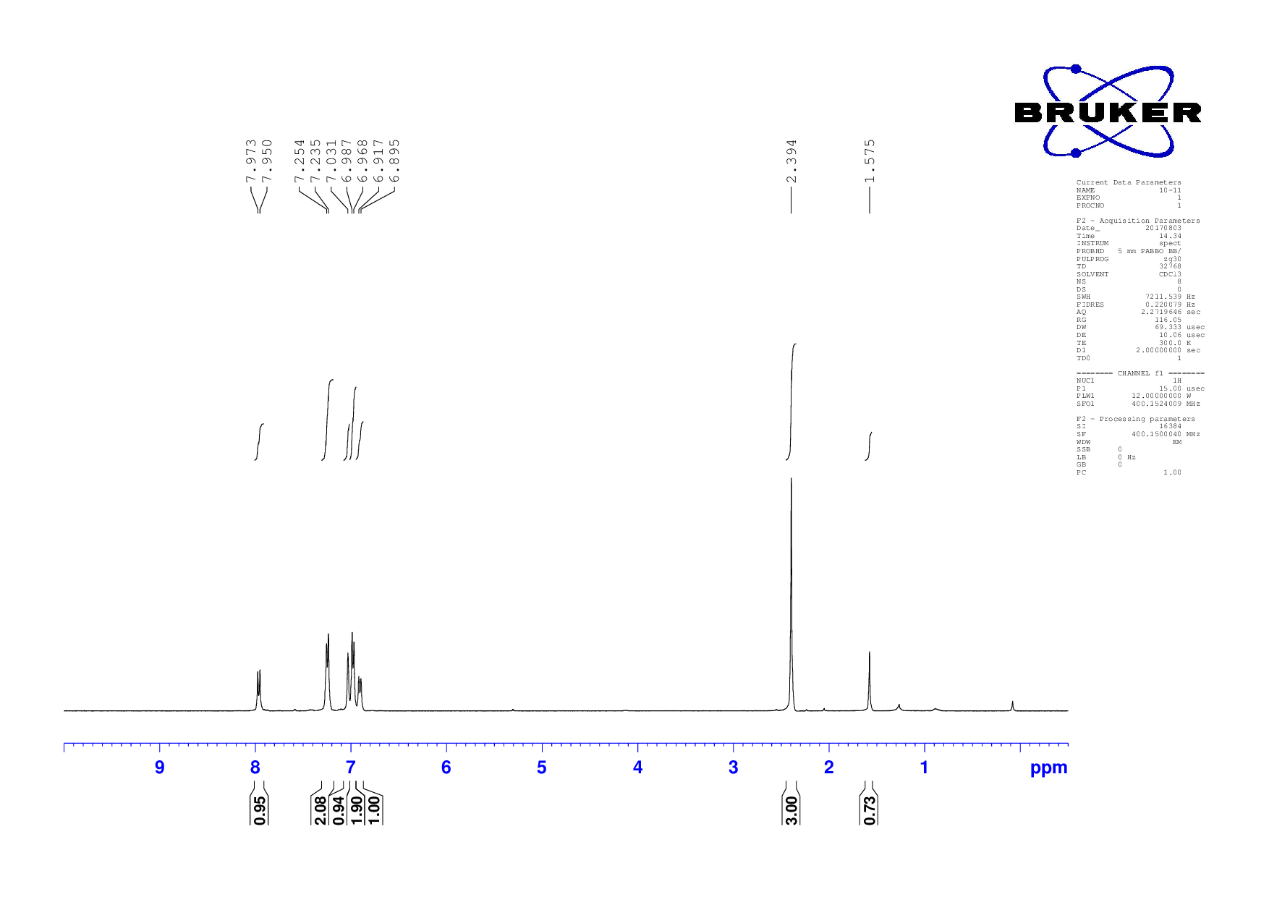


*4-fluoro-1-nitro-2-(p-tolyloxy)benzene (****5b****)*

*
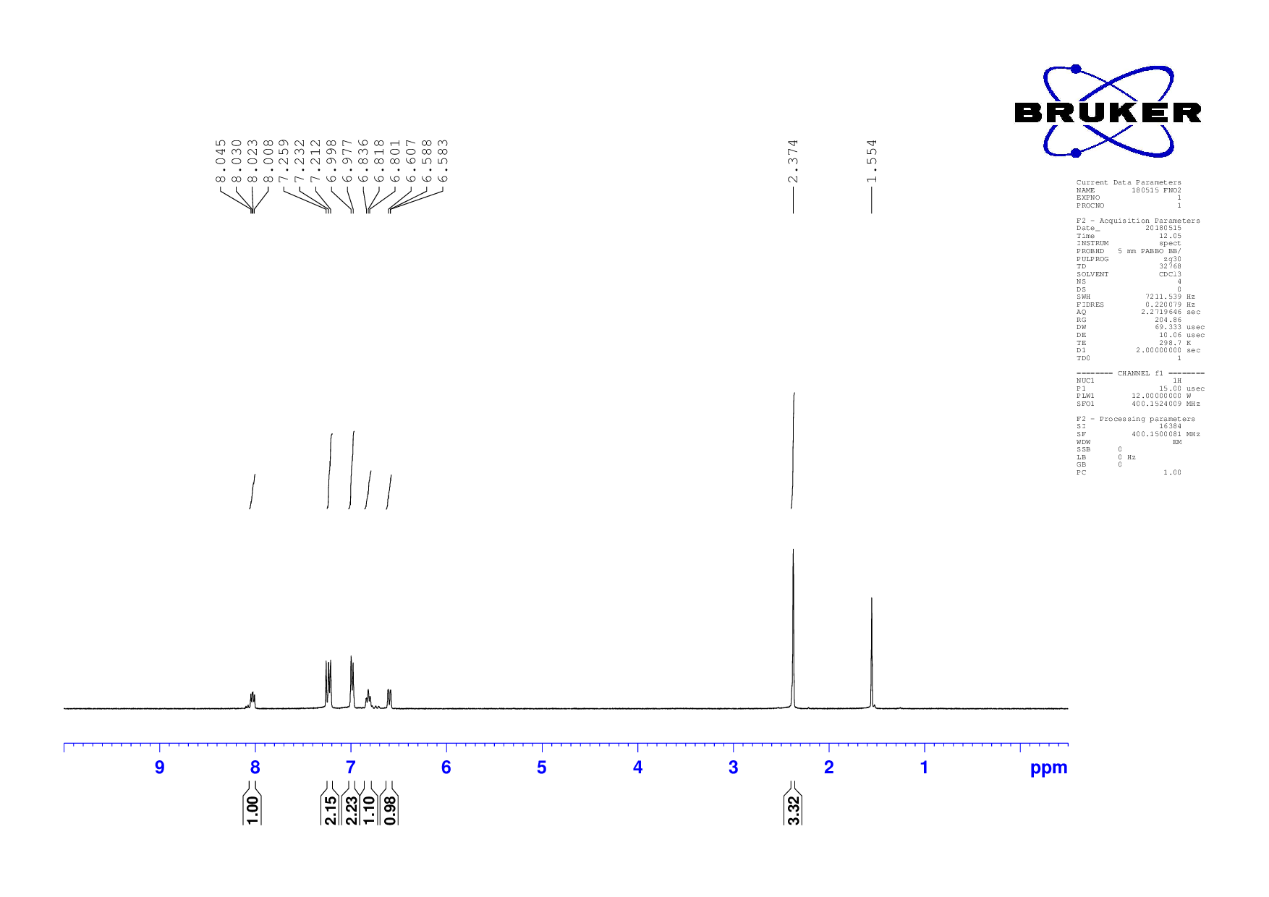
*

*
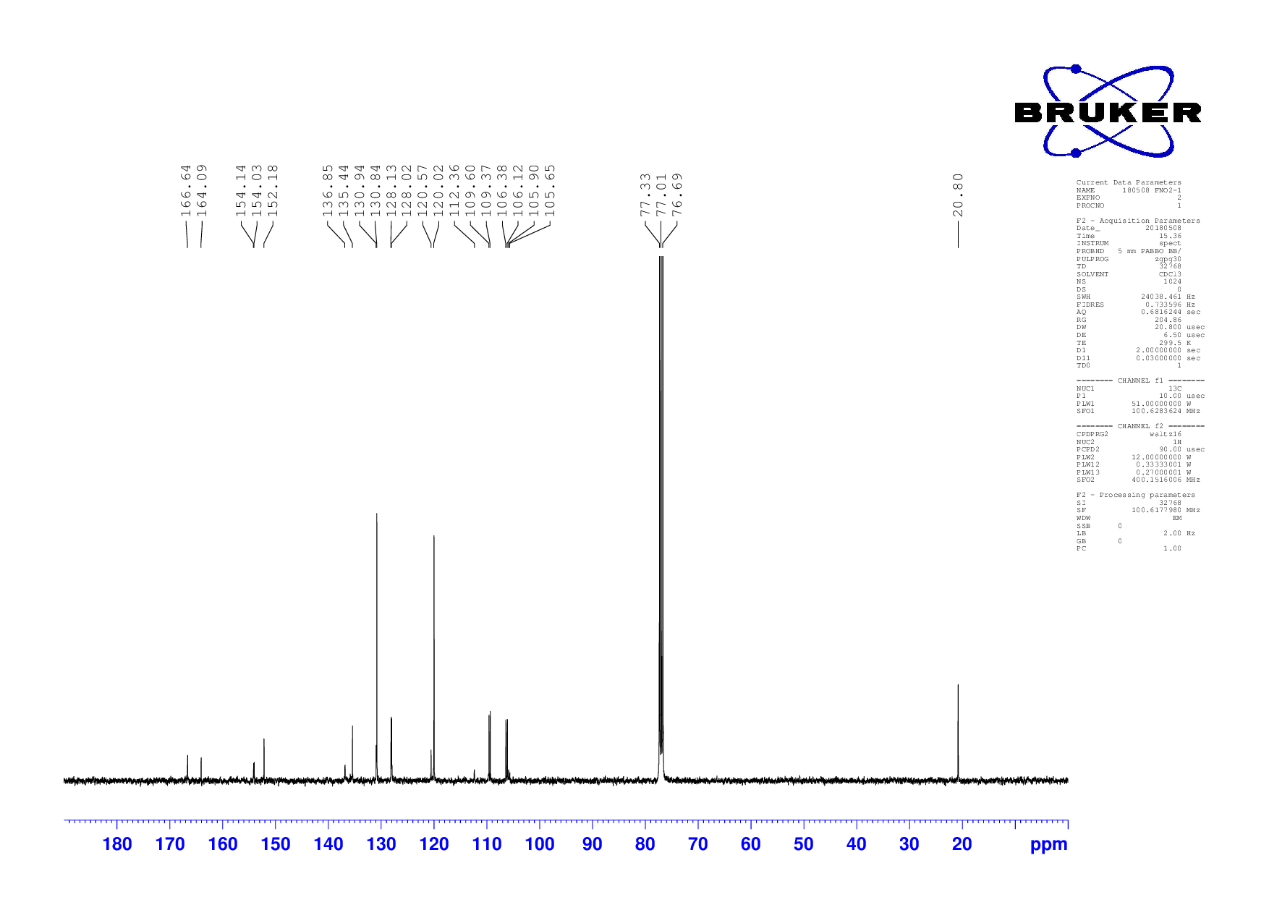
*

*4-chloro-2-(p-tolyloxy)aniline (****6a****)*

*
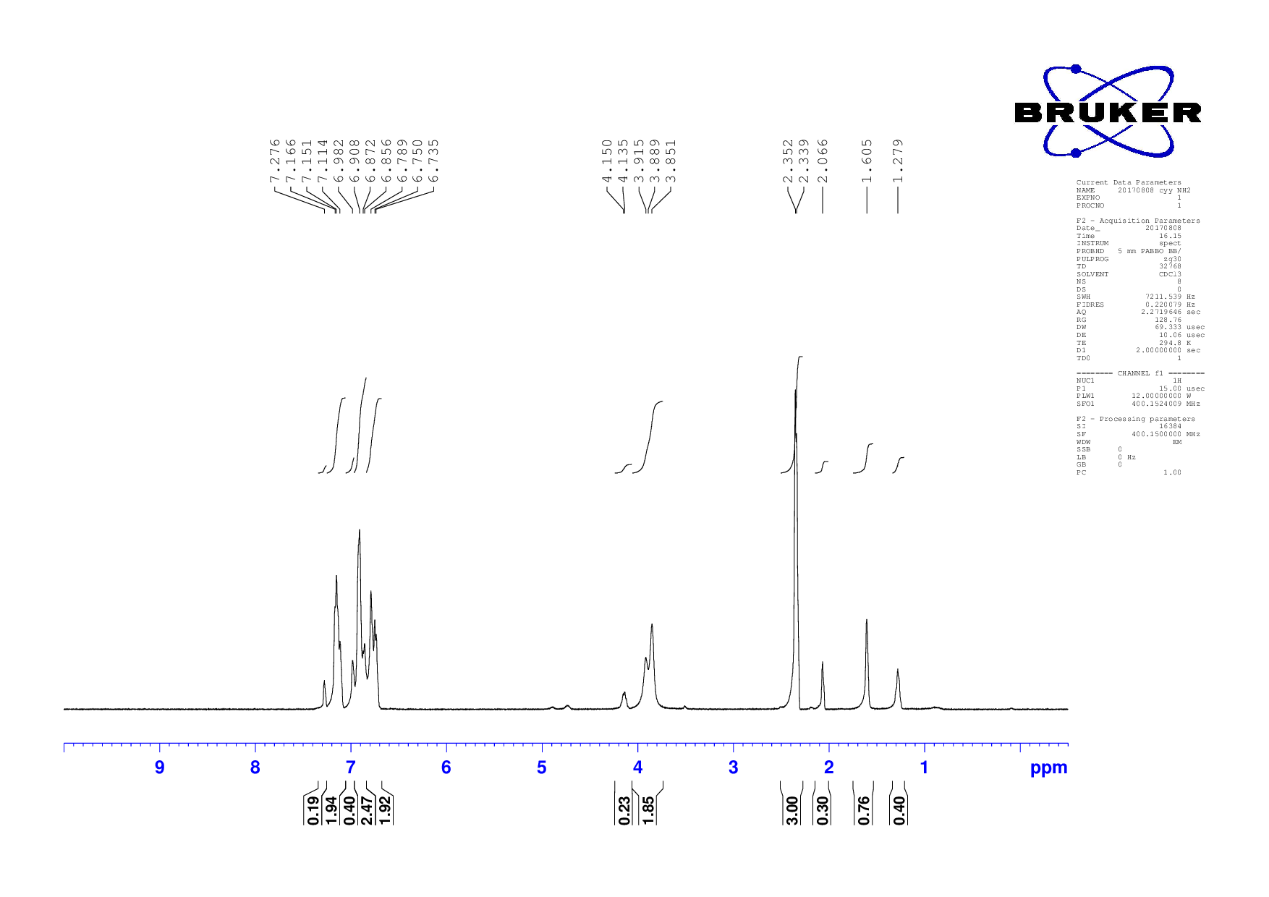
*

*4-fluoro-2-(p-tolyloxy)aniline (****6b****)*

*
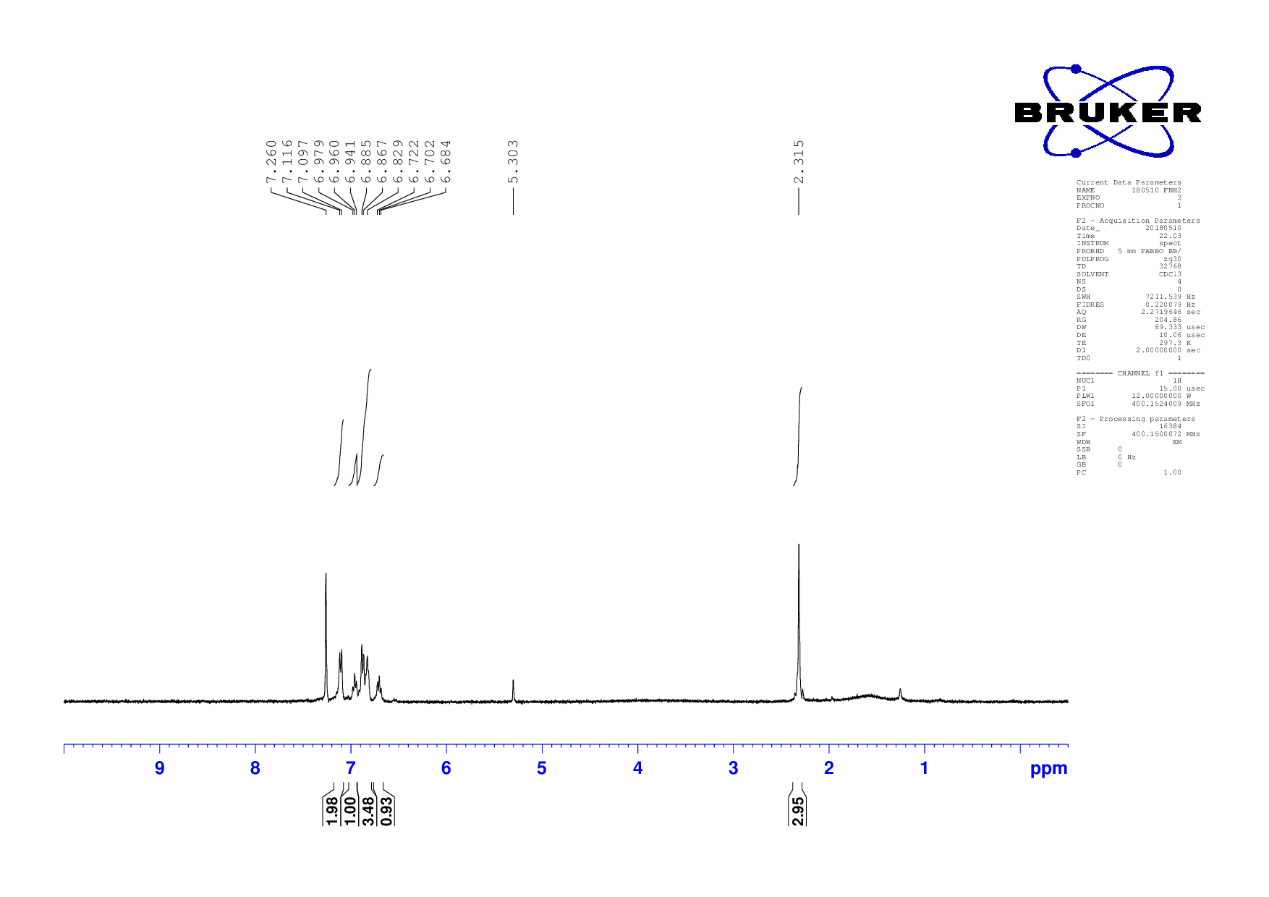
*

*2-(4-chloro-2-(p-tolyloxy)phenyl)-1,2-dihydroisoquinolin-3(4H)-one (****7****)
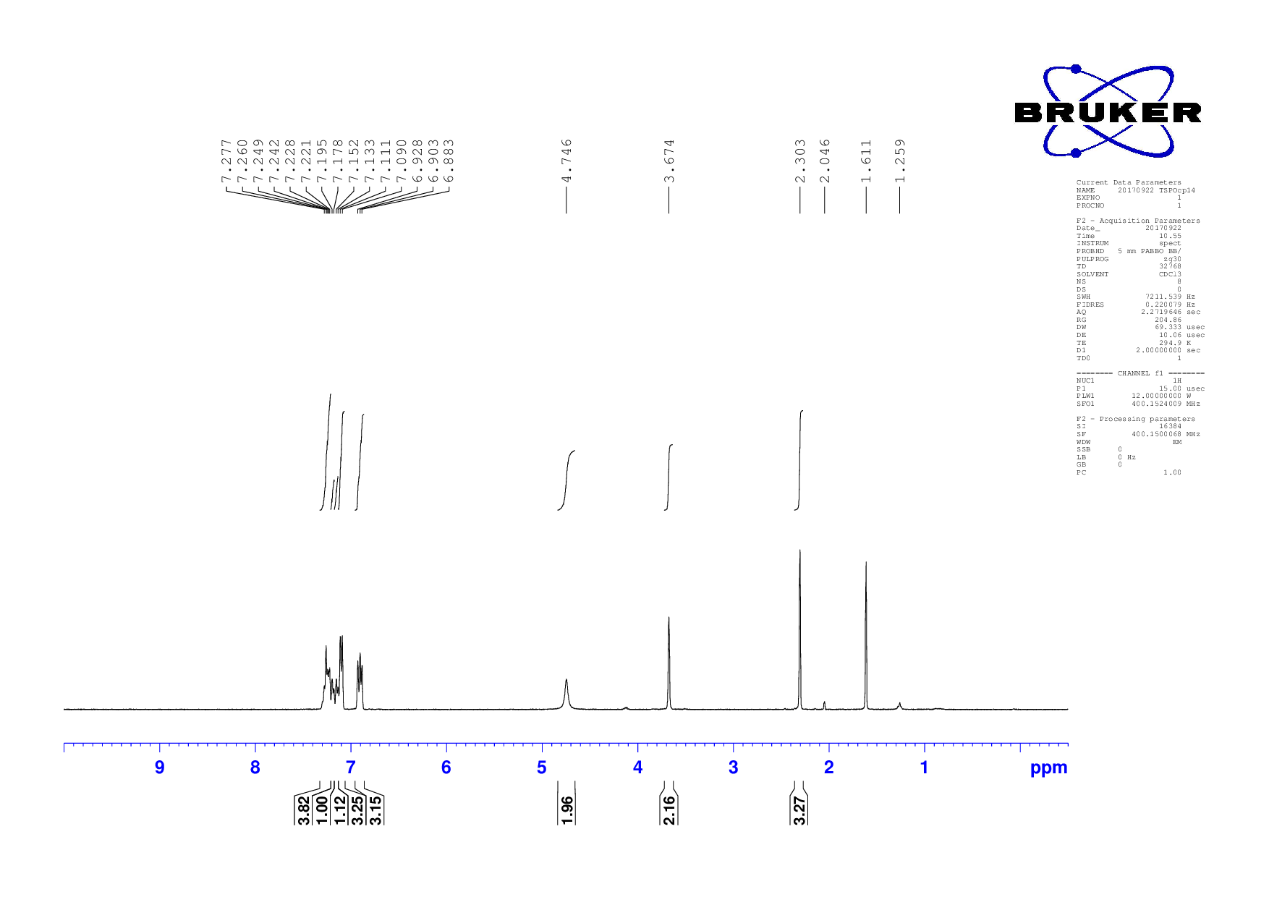
*

*
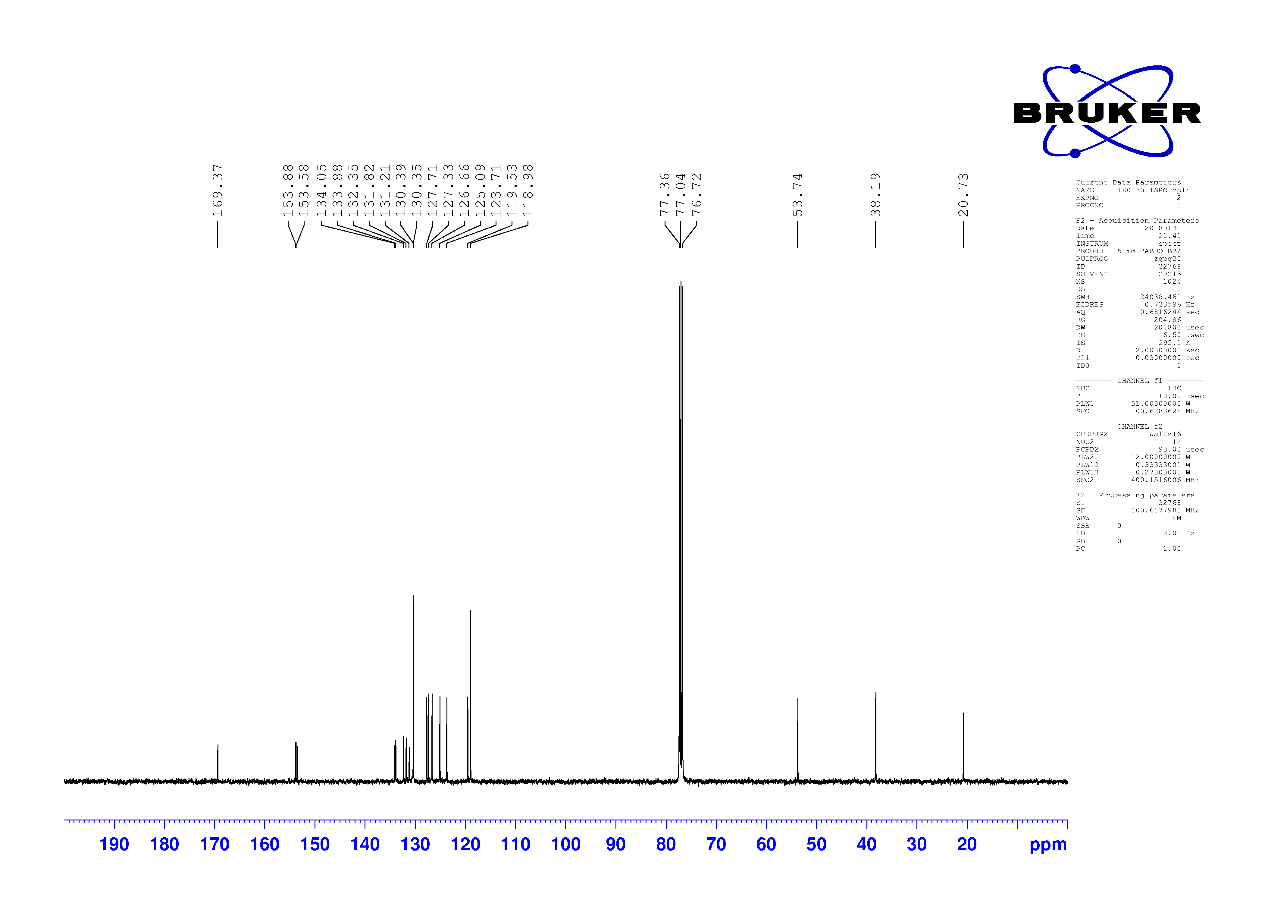
*

*2-(4-(4,4,5,5-tetramethyl-1,3,2-dioxaborolan-2-yl)-2-(p-tolyloxy)phenyl)-*

*1,2-dihydroisoquinolin-3(4H)-one (****8****)
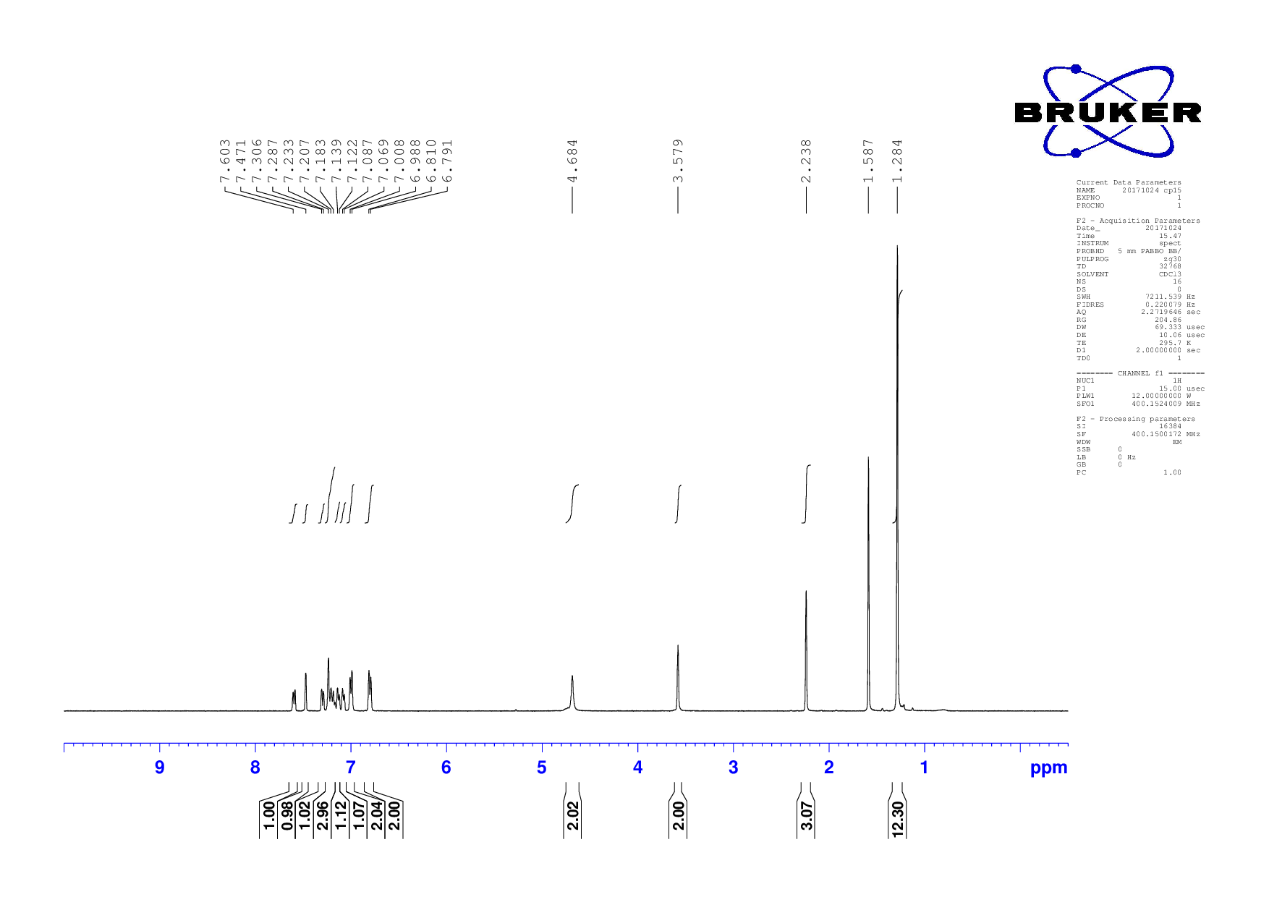

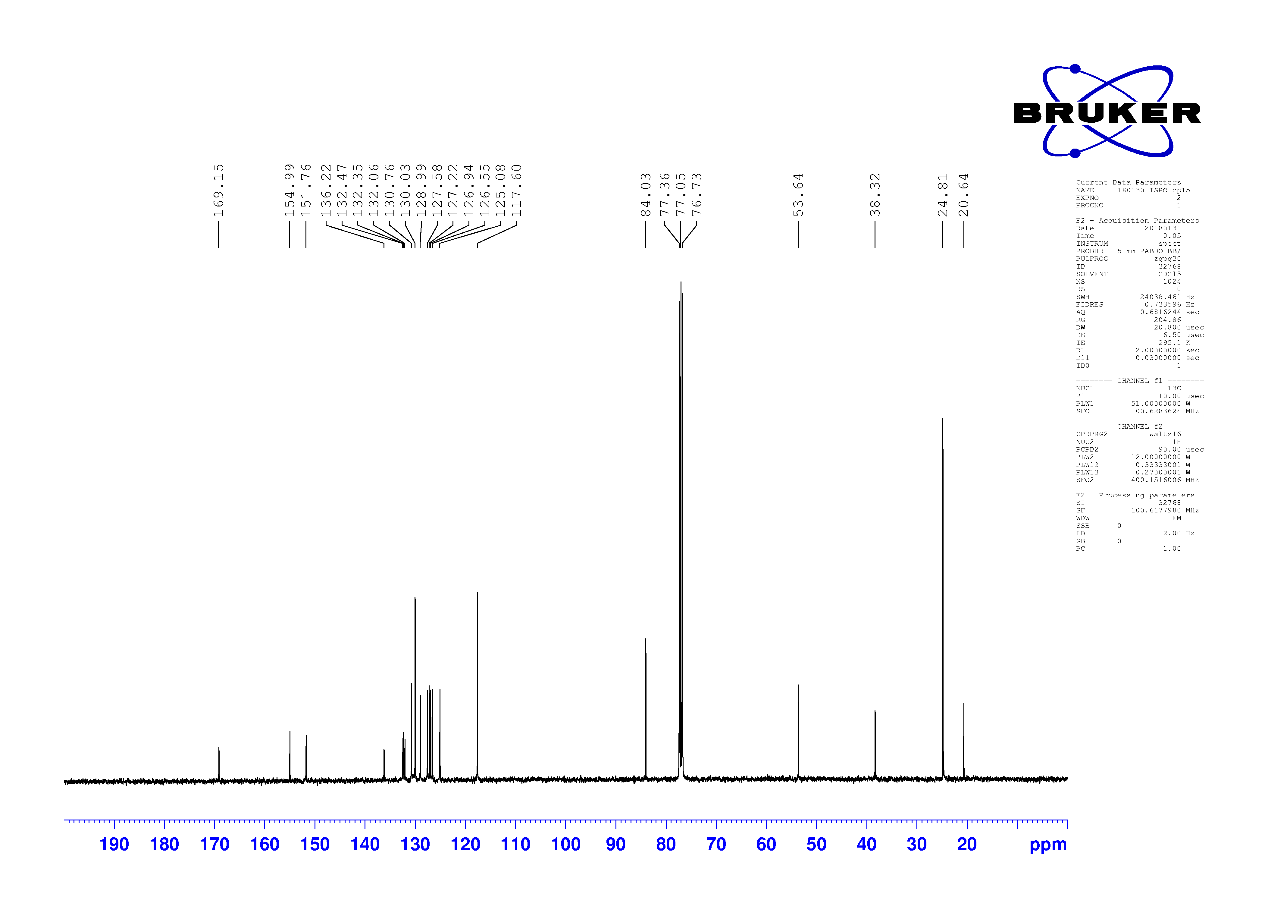
*

*
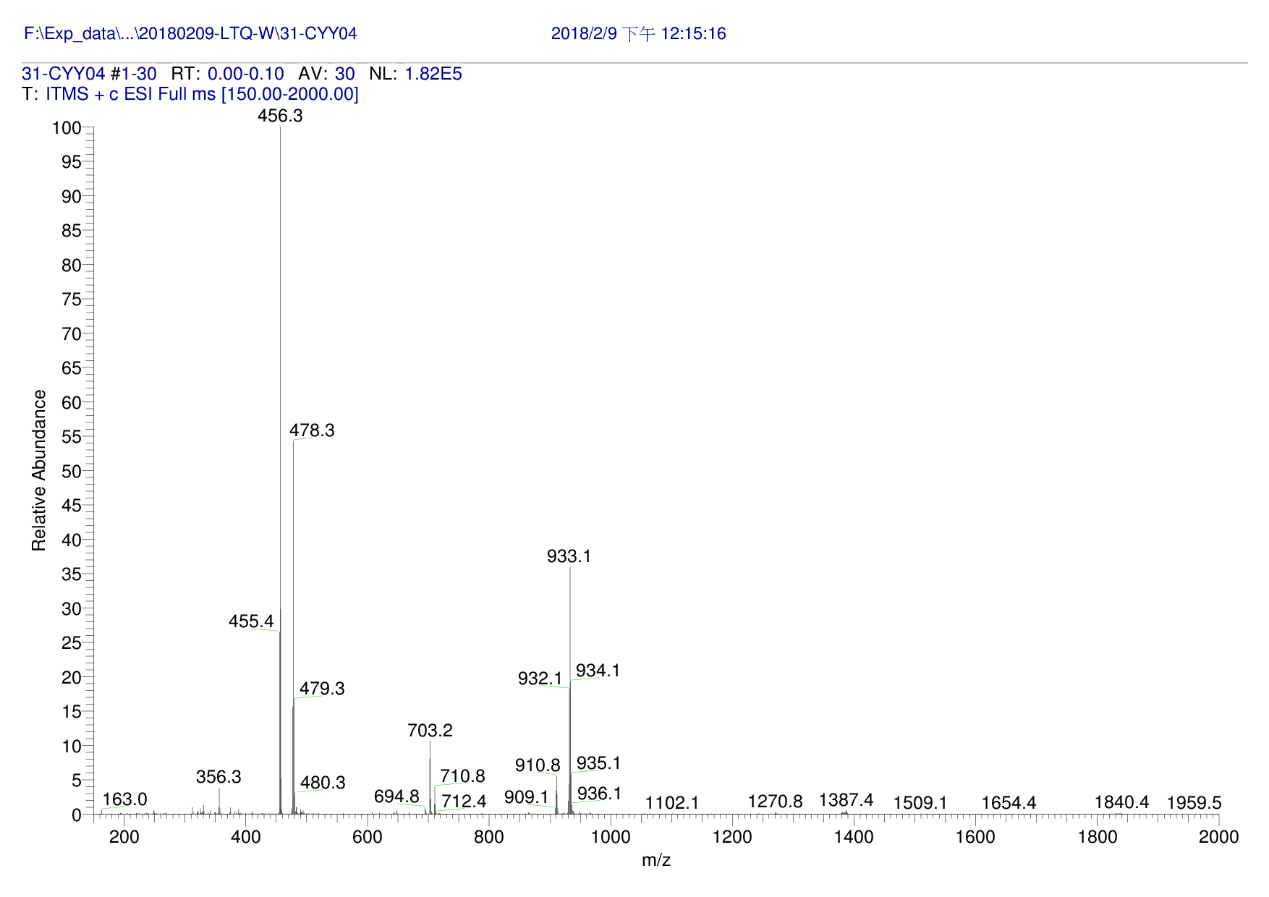
*

*2-(4-fluoro-2-(p-tolyloxy)phenyl)-1,2-dihydroisoquinolin-3(4H)-one (****10****)*

*
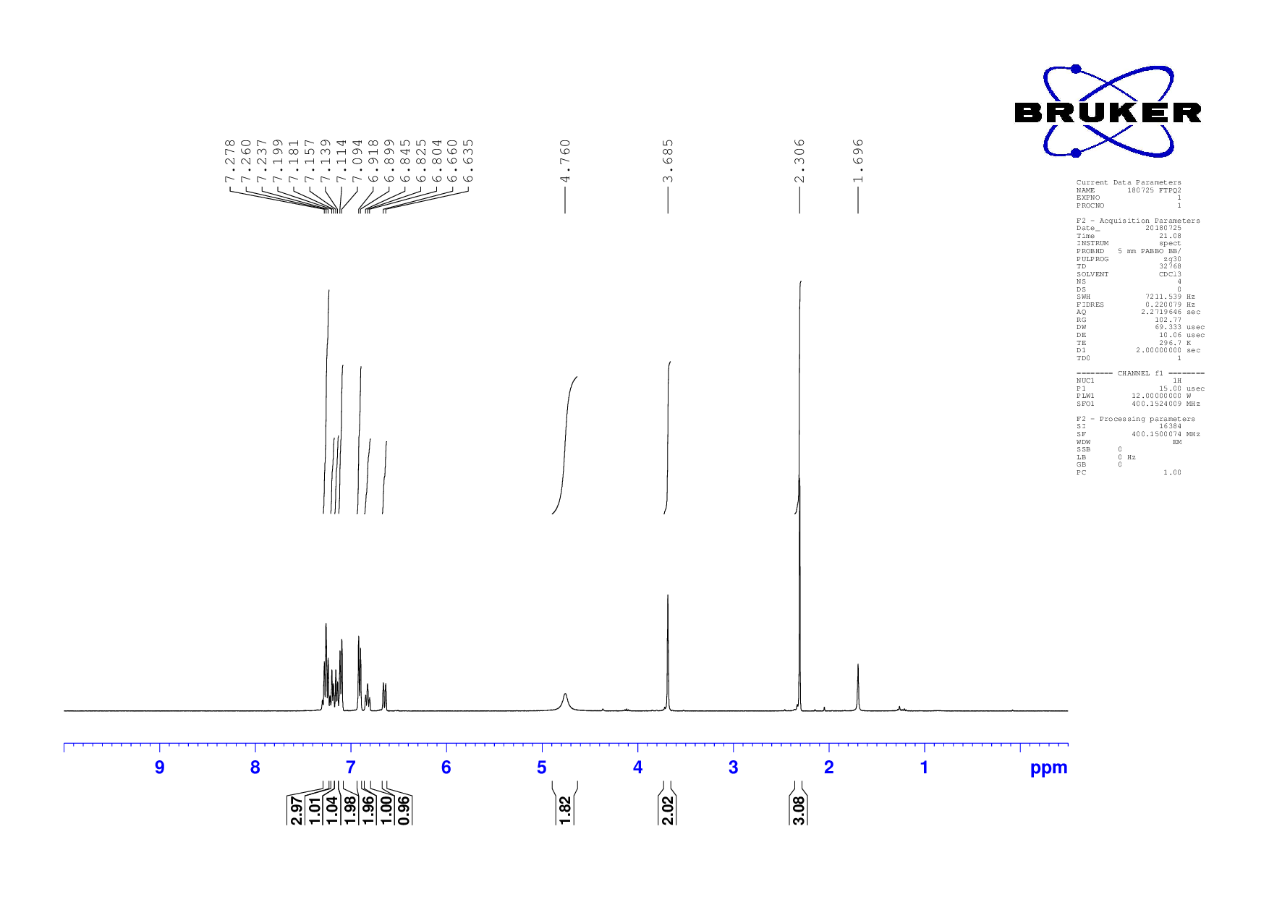
*


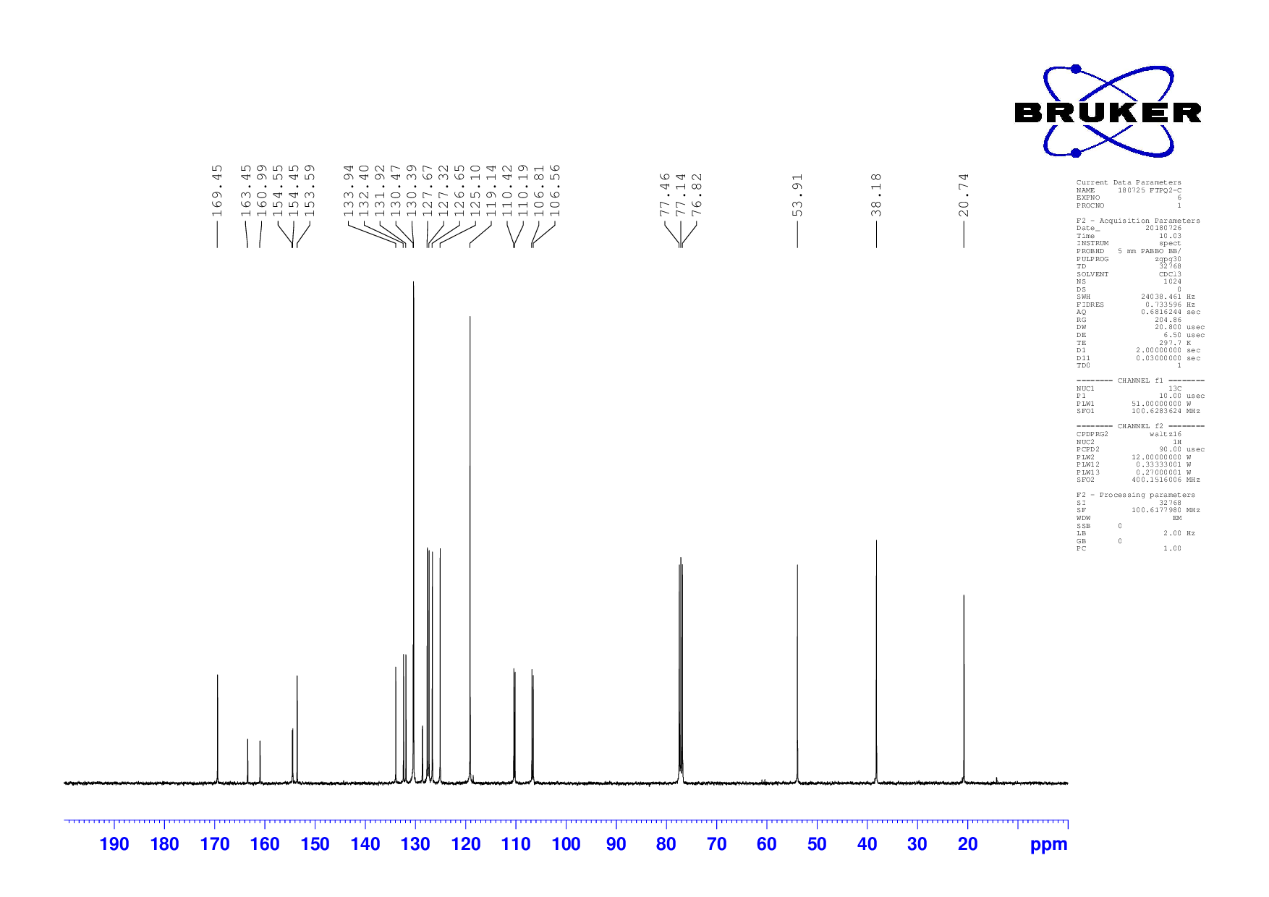


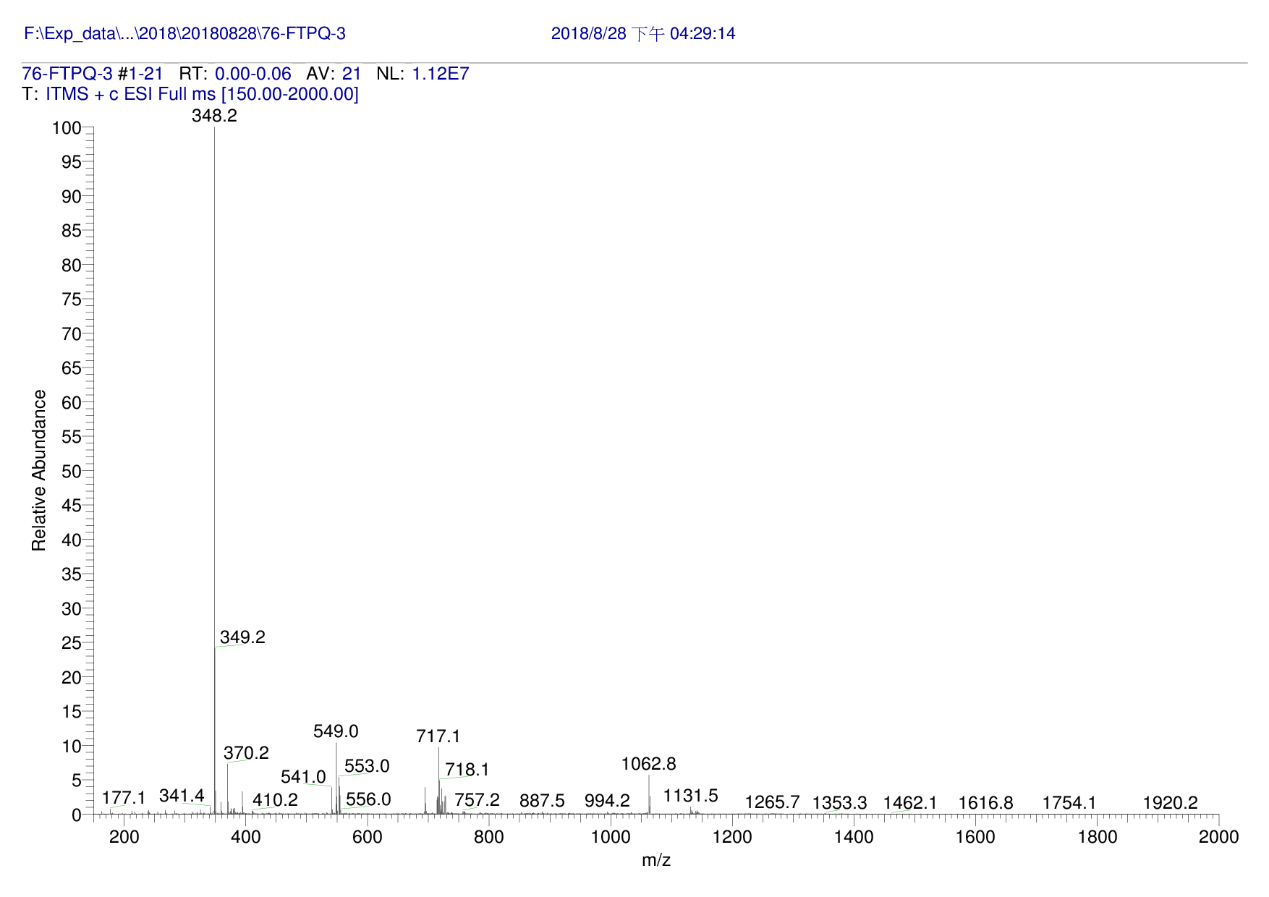


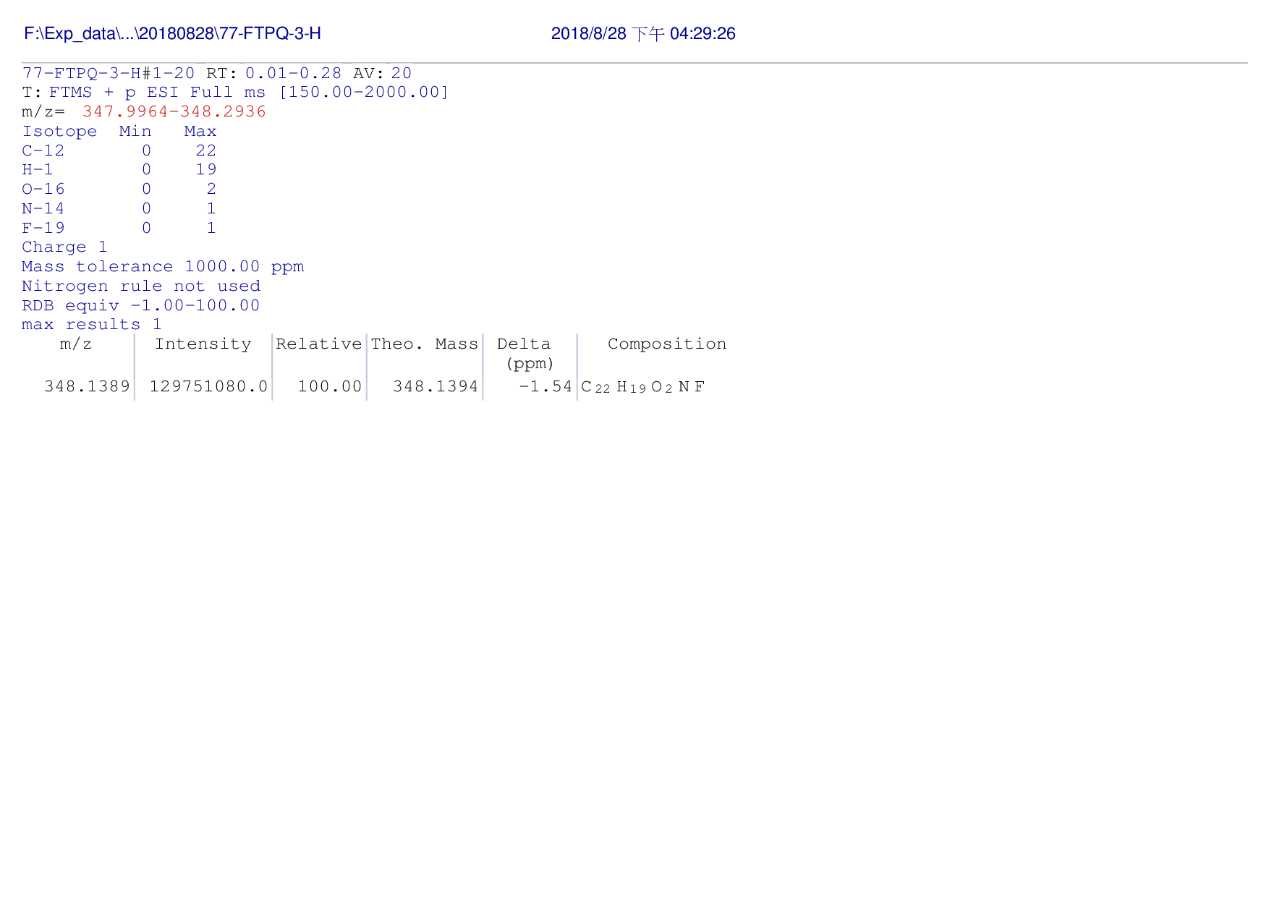

Supplement: Supplementary file 1 — All spectral data of compound 1-10. (DOCX 2913 kb) [file 12880_2019_375_MOESM1_ESM.docx]
